# Smartphone-Based Video Antenatal Preterm Birth Education: The Preemie Prep for Parents Randomized Clinical Trial

**DOI:** 10.1001/jamapediatrics.2023.1586

**Published:** 2023-07-31

**Authors:** Kathryn E. Flynn, Siobhan M. McDonnell, Ruta Brazauskas, S. Iqbal Ahamed, Jennifer J. McIntosh, Michael B. Pitt, Kris Pizur-Barnekow, U. Olivia Kim, Abbey Kruper, Steven R. Leuthner, Mir A. Basir

**Affiliations:** 1 Department of Medicine, Medical College of Wisconsin, Milwaukee; 2 Department of Pediatrics, Medical College of Wisconsin, Milwaukee; 3 Division of Biostatistics, Medical College of Wisconsin, Milwaukee; 4 Department of Computer Science, Marquette University, Milwaukee, Wisconsin; 5 Department of Obstetrics & Gynecology, Medical College of Wisconsin, Milwaukee; 6 Department of Pediatrics, University of Minnesota Masonic Children’s Hospital, Minneapolis; 7 Families First LLC, Eagle River, Wisconsin; 8 Department of Pediatrics, NorthShore University HealthSystem, Evanston, Illinois

## Abstract

**Importance:**

Preterm birth is a leading cause of infant mortality and child morbidity. Preterm birth is not always unexpected, yet standard prenatal care does not offer anticipatory education to parents at risk of delivering preterm, which leaves parents unprepared to make health care choices during the pregnancy that can improve survival and decrease morbidity in case of preterm birth.

**Objective:**

To evaluate the effect of the Preemie Prep for Parents (P3) program on maternal knowledge of preterm birth, preparation for decision-making, and anxiety.

**Design, Setting, and Participants:**

Recruitment for this randomized clinical trial conducted at a US academic medical center took place from February 3, 2020, to April 12, 2021. A total of 120 pregnant persons with a risk factor for preterm birth were enrolled between 16 and 21 weeks’ gestational age and followed up through pregnancy completion.

**Intervention:**

Starting at 18 weeks’ gestational age, P3 program participants received links delivered via text message to 51 gestational age–specific short animated videos. Control participants received links to patient education webpages from the American College of Obstetricians and Gynecologists.

**Main Outcomes and Measures:**

At 25 weeks’ gestation, scores on the Parent Prematurity Knowledge Questionnaire (scored as percent correct), Preparation for Decision Making Scale (scored 0-100), and Patient-Reported Outcomes Measurement Information System (PROMIS) Anxiety computerized adaptive test. Analysis was based on an intention to treat.

**Results:**

A total of 120 pregnant participants (mean [SD] age, 32.5 [4.9] years) were included in the study; 60 participants were randomized to each group. Participants in the P3 group scored higher than those in the control group on knowledge of long-term outcomes at 25 weeks (88.5% vs 73.2%; estimated difference, 15.3 percentage points; 95% CI, 8.3-22.5 percentage points; *P* < .001). Participants in the P3 group reported being significantly more prepared than did participants in the control group for neonatal resuscitation decision-making at 25 weeks (Preparation for Decision Making Scale score, 76.0 vs 52.3; difference, 23.7; 95% CI, 14.1-33.2). There was no difference between the P3 group and the control group in anxiety at 25 weeks (mean [SE] PROMIS Anxiety scores, 53.8 [1.1] vs 54.0 [1.1]; difference, −0.1; 95% CI, −3.2 to 2.9).

**Conclusions and Relevance:**

In this randomized clinical trial, pregnant persons randomly assigned to the P3 program had more knowledge of core competencies and were more prepared to make decisions that affect maternal and infant health, without experiencing worse anxiety. Mobile antenatal preterm birth education may provide a unique benefit to parents with preterm birth risk factors.

**Trial Registration:**

ClinicalTrials.gov Identifier: NCT04093492

## Key Points

**Question:**

What are the effects of a smartphone-based preterm birth education program in early pregnancy on recommended parental preterm birth knowledge, preparation for decision-making, and parental anxiety?

**Finding:**

In a parallel-group randomized clinical trial of 120 pregnant participants with a risk factor for preterm birth, those assigned to the smartphone preterm birth video education program (intervention group) had more knowledge of core competencies and were more prepared to make decisions that affect maternal and infant health, without experiencing increased anxiety, compared with participants assigned to the control group.

**Meaning:**

Anticipatory preterm birth education in early pregnancy may empower parents with known preterm birth risk factors to participate in medical care decisions in the event of preterm birth.

## Teaser

This randomized clinical trial evaluates the effect of the Preemie Prep for Parents program on parental knowledge of preterm birth, preparation for decision-making, and parent anxiety.

## Introduction

A term pregnancy is 40 weeks’ gestational age (GA), and birth at or before 36 weeks’ GA is considered preterm.^1^ In the US, a preterm infant is born almost every minute.^2^ Despite modern neonatal intensive care unit (NICU) treatments and irrespective of GA at birth, preterm infants experience more death and disability than full-term infants.^3,4^ Preterm birth is the leading cause of US infant mortality and child morbidity.^5^ Several prenatal health care decisions improve survival and decrease morbidity in preterm infants, including delivering at a risk-appropriate birth hospital^6,7^ and deciding to breastfeed.^8^ Preterm birth is not always unexpected. Half of the 500 000 US mothers who deliver preterm infants receive diagnoses of risk factors months before the delivery,^9^ yet education on preterm birth and medical decision-making is not consistently provided to them until the delivery hospitalization^10^ due to clinicians’ concern that it will increase parental anxiety.^11^

Providing preterm birth education during the delivery hospitalization, when the pregnancy is ending, misses the opportunity to influence pregnancy outcomes. It does not allow the family time to contemplate and discuss the presented information, especially for resuscitation decision-making for extremely premature infants.^9^ To bridge this gap in prenatal education and support wider dissemination of information to parents at high risk, we developed a multimedia educational aid, the Preemie Prep for Parents (P3) program, in collaboration with parents of preterm infants and perinatal clinicians and based on published guidelines.^12,13,14^ Smartphone-based education allows for learning in small increments at a time and place that suits the patient. Short, animated videos have broad appeal^15,16^ and educational efficacy among patients with lower health literacy.^17^ Moreover, previous work has found higher anxiety among patients hospitalized for preterm labor^18^ but also found that targeted education reduces anxiety.^19^ Thus, we hypothesized that, compared with static patient education webpages (control), the P3 program would improve recommended parental preterm birth knowledge, improve preparation for decision-making, and would not increase parental anxiety.

## Methods

This was a parallel-group randomized clinical trial conducted at an academic medical center near Milwaukee, Wisconsin. All participants received standard prenatal care. The intervention group received the P3 program, and the control group received links to patient education webpages. This study was approved by the Medical College of Wisconsin institutional review board, and all participants gave written informed consent. As pregnant minors act as decision-makers through pregnancy and preterm birth, we had institutional review board approval to enroll pregnant minors aged 13 to 17 years without additional parental consent (trial protocol in Supplement 1). This study followed the Consolidated Standards of Reporting Trials (CONSORT) reporting guideline.

### P3 Program

Content for the P3 program was created based on the information recommended for parents at risk of preterm birth by the Society for Maternal-Fetal Medicine (SMFM), American Academy of Pediatrics (AAP), and American College of Obstetricians and Gynecologists (ACOG).^14^ This content was integrated with the results of a pilot study^13^ and a previous focus group with parents who had experienced preterm birth.^12^ Priorities included the risk-appropriate level of NICU, importance of breastfeeding, periviable birth treatment options, and factors other than GA that affect outcome. Learning objectives were developed into brief video scripts that were refined in a collaborative and iterative process and incorporated feedback from medical professionals and parent advisors. Fifty-one animated videos were created using the software Powtoon (Powtoon.com Inc), with professional voice actors providing narration (Video). Additional refinements were made through usability testing (eMethods in Supplement 2). The videos ranged in length from 1 to 3 minutes.

**Video.** Example Preemie Prep for Parents (P3) Video About Antenatal Corticosteroids This example of a P3 video (.mp4) introduces the purpose and recommended dosage of antenatal corticosteroids, highlighting the value of early medical care to receive the optimum dosage.

The control group was provided links by email or text message to patient education webpages created by the ACOG. These publicly available webpages featured information about frequently asked questions on preterm labor, extremely preterm birth, cesarean birth, and a partner’s guide to pregnancy.

### Participants and Procedures

Study recruitment occurred in person from February 3 to March 13, 2020, and, due to the COVID-19 pandemic, by telephone from April 6, 2020, to April 12, 2021. Pregnant persons aged 13 years or older with a risk factor for preterm birth were approached prior to a prenatal appointment at a maternal-fetal medicine clinic and enrolled between 16 weeks plus 0 days’ GA and 21 weeks plus 6 days’ GA. Risk factors included a short cervix, multifetal gestation, history of spontaneous preterm birth, preeclampsia (at ≤34 weeks’ GA), chronic hypertension, diabetes requiring medications, and intrauterine growth restriction. Those unable to speak English and pregnancies with significant birth defects were excluded. Participants were followed up through pregnancy completion. Final data were collected in July 2021.

After consent in Research Electronic Data Capture (REDCap),^20^ participants completed an enrollment questionnaire and were randomized 1:1 to the P3 (intervention) or control group. To ensure balanced groups in GA at the time of enrollment, randomization was stratified into 2 blocks: 16 to 18 weeks’ GA and 19 to 21 weeks’ GA. The randomization list was generated using the R package blockrand (R Group for Statistical Computing) and implemented through REDCap. Participants randomized between 16 and 18 weeks’ GA began receiving text messages at 18 weeks’ GA, and participants randomized between 19 and 21 weeks’ GA began receiving text messages at enrollment. Participants in the P3 group were sent 0 to 2 text messages per day (schedule dependent on GA at enrollment), which linked to the GA-specific videos. Participants in the control group received an email or text message, depending on their preference, with links to 6 ACOG webpages. We collected clinical data from the medical record regarding the GA at delivery.

### Patient-Reported Outcome Assessments

The self-administered baseline assessment included de novo items on sociodemographic characteristics, perceived likelihood of preterm birth, preference for involvement in medical decisions, and typical smartphone use. Race and ethnicity were self-reported using National Institutes of Health–specified racial and ethnic categories. Scores of several previously validated scales were also collected: the Decision Self-Efficacy Scale,^21^ an 11-item measure of confidence in one’s abilities to participate in decision-making; the Patient-Reported Outcomes Measurement Information System (PROMIS) Anxiety Computerized Adaptive Testing (CAT),^22^ which measures general anxiety over the past 7 days; the PROMIS Global-10,^23^ a global assessment of physical, mental, and social health; and the Brief Health Literacy Screen.^24,25^ For PROMIS measures, scores were converted to a normed T score, where a mean (SD) of 50 (10) corresponds to the US general population mean.

If pregnancy continued, follow-up assessments were administered at 25, 30, and 34 weeks’ GA. Participants were given $20 for each completed assessment. Questionnaires were automatically delivered by REDCap via text message or email, depending on participant preference. Follow-up assessments included the PROMIS Anxiety CAT, the Preparation for Decision Making Scale, and the Parent Prematurity Knowledge Questionnaire (PPKQ). The Preparation for Decision Making Scale is a 10-item measure of how well a decision aid prepared someone to communicate with clinicians about a health care decision. It has been shown to discriminate between patients who did and patients who did not find a decision aid helpful, with a difference of 12 points on a 100-point scale and SD of 20.^26^ It is designed to be customized to a particular decision: at 25 weeks, we asked about resuscitation decisions for periviable birth; at 30 weeks, we asked about a risk-appropriate choice of birth hospital; and at 34 weeks, we asked about the decision to breastfeed. We developed the PPKQ to measure the core competencies needed to participate in decisions about preterm birth. Items were drafted by a multidisciplinary team of experts (K.E.F., J.J.M., K.P-B., U.O.K., and M.A.B), with content selected based on SMFM-, AAP-, and ACOG-recommended information. Items regarding resuscitation decisions were concordant with the policies of the hospital system from which participants were recruited. Items were evaluated in conjunction with the video usability testing and iteratively refined in multiple rounds of cognitive interviews with diverse parents.^27,28^ The PPKQ includes an overall score and 8 subscale scores representing knowledge in the SMFM-, AAP-, and ACOG-recommended domains: (1) long-term outcomes, (2) variability in due date estimation, (3) general prematurity knowledge, (4) lowest GA needed for survival, (5) factors influencing preterm birth outcome, (6) treatment options, (7) short-term outcomes, and (8) advocacy. The long-term outcomes subscale of the PPKQ was selected as the primary outcome because parent knowledge of long-term outcomes is often worse than short-term outcomes^19^ despite the centrality of long-term outcomes in periviable decision-making.^29^ The PPKQ can be scored as a sum or percentage correct. The 25-week instrument is available (eMethods in Supplement 2). At 34 weeks’ GA (or at preterm birth), participants were additionally asked for feedback regarding the study materials they received.

### Tracking Video Use

Tracking software was developed to record participant use of the P3 program. The software tracked the videos sent, the links participants clicked, the videos played, and the time played for each viewing.

### Statistical Analysis

We powered the study based on an expected mean (SD) of 50% (35%) correct on the PPKQ long-term outcomes subscale in the control group at 25 weeks. With power of 80% and α of .05, we needed 50 participants in each group to detect a 20% difference in knowledge scores for the P3 group. To allow for attrition and preterm deliveries, we enrolled 120 pregnant participants.

We conducted blinded analysis with masked group assignment in the data set. Mean (SD) values were used to summarize continuous variables, while counts and percentages were used for categorical variables. The prespecified intention-to-treat analysis used linear regression to compare pregnant participants in the 2 study groups on PPKQ long-term outcomes at 25 weeks while adjusting for GA at enrollment. Analysis of secondary outcomes was not adjusted for stratification. We used all available data without imputing missing values. We used linear regression to examine associations between knowledge and P3 use captured by the tracking software. All statistical tests were 2-sided; statistical analyses were conducted using SAS, version 9.4 (SAS Institute Inc).

## Results

**Figure 1.**
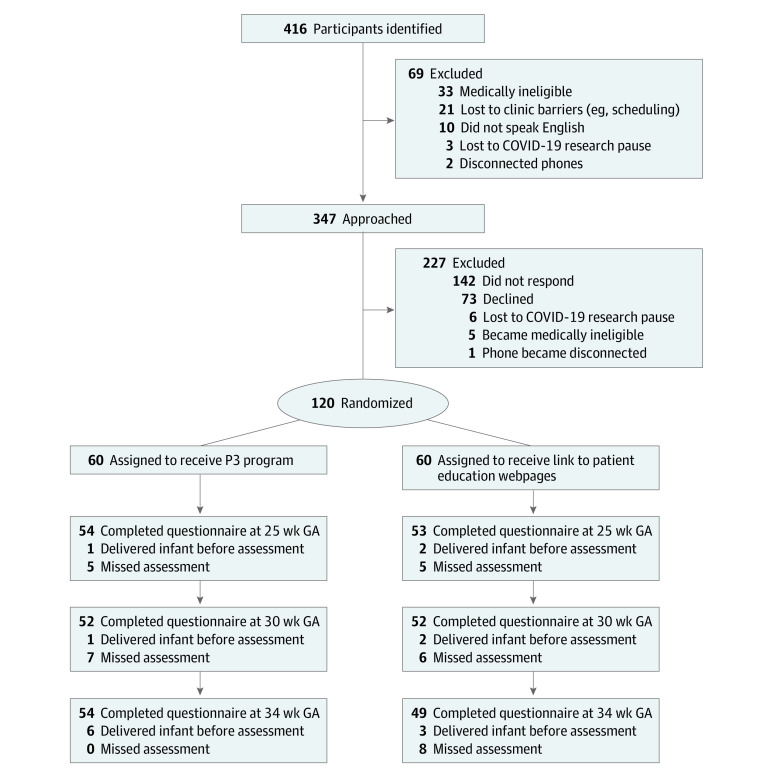
CONSORT Diagram GA indicates gestational age; P3, Preemie Prep for Parents program.

We enrolled 120 pregnant patients (mean [SD] age, 32.5 [4.9] years), with 60 randomly assigned to each group (Figure 1). One participant was lost to follow-up. Missing data due to questionnaire nonresponse were 8.3% (10 of 120) at 25 weeks, 10.8% (13 of 120) at 30 weeks, and 6.7% (8 of 120) at 34 weeks.

There were no differences in the baseline characteristics of participants by study group (Table 1), indicating successful randomization. Although the US population rate of preterm birth is 10%, in our study 46 of 119 of the participants (38.7%) delivered preterm, indicating successful intervention targeting by risk factor (eTable 1 in Supplement 2).

**Table 1.** Pregnant Participant Characteristics at Baseline

Characteristic	Total (N = 120)	P3 group (n = 60)	Control group (n = 60)
Race, No. (%)			
American Indian	1 (0.8)	0	1 (1.7)
Asian	5 (4.2)	1 (1.7)	4 (6.7)
Black	29 (24.2)	15 (25.0)	14 (23.3)
White	89 (74.2)	47 (78.3)	42 (70.0)
Other^a^	1 (0.8)	1 (1.7)	0
Ethnicity, No./total No. (%)			
Hispanic or Latino	11/119 (9.2)	4/59 (6.8)	7 (11.7)
Not Hispanic or Latino	108/119 (90.8)	55/59 (93.2)	53 (88.3)
Missing, No.	1	1	0
Age group, No. (%)			
13-19 y	0	0	0
20-24 y	10 (8.3)	5 (8.3)	5 (8.3)
25-34 y	74 (61.7)	38 (63.3)	36 (60.0)
35-46 y	36 (30.0)	17 (28.3)	19 (31.7)
Educational level, No. (%)			
9th-12th Grade, no diploma	2 (1.7)	0	2 (3.3)
High school diploma	18 (15.0)	8 (13.3)	10 (16.7)
Some college	22 (18.3)	13 (21.7)	9 (15.0)
2-y Degree	15 (12.5)	7 (11.7)	8 (13.3)
4-y Degree	33 (27.5)	17 (28.3)	16 (26.7)
Graduate or professional degree	30 (25.0)	15 (25.0)	15 (25.0)
Marital status, No./total No. (%)			
Married	77/119 (64.7)	38/59 (64.4)	39 (65.0)
Not married	42/119 (35.3)	21/59 (35.6)	21 (35.0)
Missing, No.	1	1	0
Living situation, No./total No. (%)			
Living with infant’s father	105/119 (88.2)	54/59 (91.5)	51 (85.0)
Living separately	14/119 (11.8)	5/59 (8.5)	9 (15.0)
Missing, No.	1	1	0
GA at enrollment, wk			
16	32 (26.7)	17 (28.3)	15 (25.0)
17	22 (18.3)	13 (21.7)	9 (15.0)
18	11 (9.2)	2 (3.3)	9 (15.0)
19	17 (14.2)	10 (16.7)	7 (11.7)
20	27 (22.5)	12 (20.0)	15 (25.0)
21	9 (7.5)	5 (8.3)	4 (6.7)
22^b^	2 (1.7)	1 (1.7)	1 (1.7)
Preterm birth risk factor(s)^c^			
Chronic hypertension	26 (21.7)	10 (16.7)	16 (26.7)
Diabetes requiring medications	17 (14.2)	7 (11.7)	10 (16.7)
Fetal growth restriction	0	0	0
History of preeclampsia	11 (9.2)	2 (3.3)	9 (15.0)
History of preterm birth	47 (39.2)	21 (35.0)	26 (43.3)
Shortened cervix	5 (4.2)	2 (3.3)	3 (5.0)
Multiple births	39 (32.5)	23 (38.3)	16 (26.7)

Abbreviations: GA, gestational age; P3, Preemie Prep for Parents.

^a^ Self-reported as “other” by participant.

^b^ Two participants consented at 21 weeks’ GA but did not complete enrollment until they began 22 weeks’ GA.

^c^ Since participants may have more than 1 of the preterm birth risk factors, these totals may not add up to 100%.

Participants in the P3 group watched a mean (SD) of 54.9% (35.1%) of the videos over the 16-week intervention (eFigure in Supplement 2). Use differed by video, with a range from 33.3% to 88.3% of participants accessing each video; videos sent earlier in the pregnancy were used more often (eResults in Supplement 2). Rates of video use differed by a few important participant characteristics (eTable 2 in Supplement 2), with higher use among married participants and those with more education and lower use among Black participants and those with a history of preterm birth. Video use did not differ by ethnicity, GA at enrollment, or self-reported daily telephone screen time (eTable 2 in Supplement 2).

### Prematurity Knowledge

**Figure 2.**
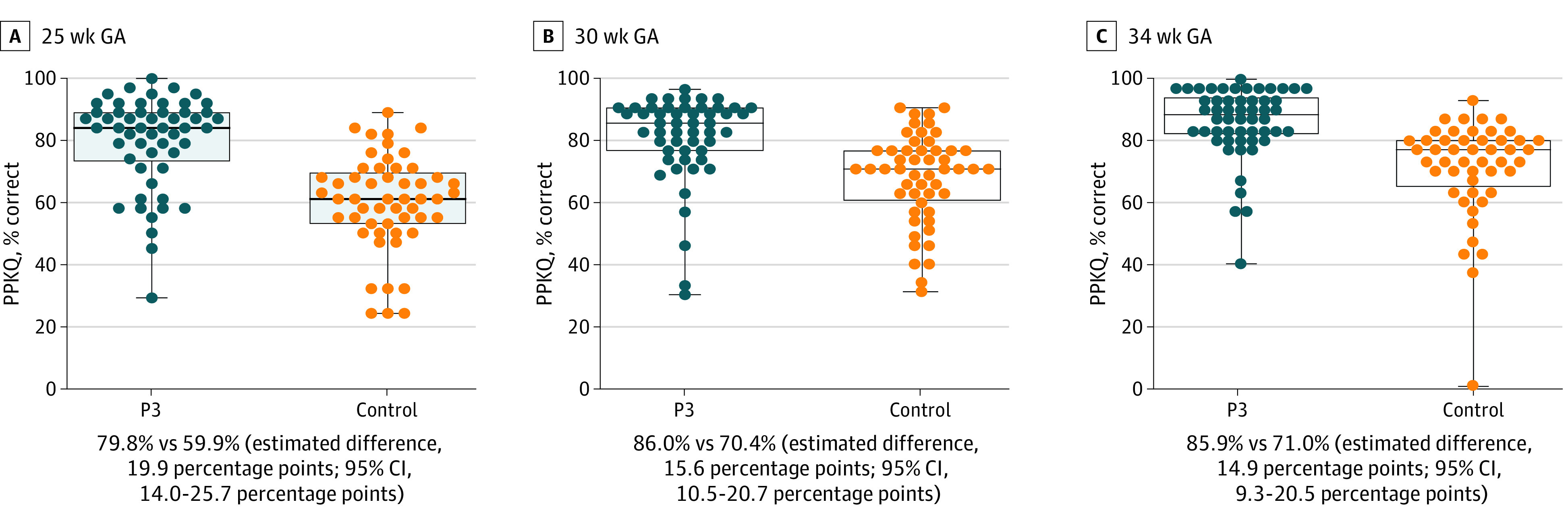
Parent Prematurity Knowledge Questionnaire (PPKQ) Scores by Study Group Reported as mean percentage of correct responses with the estimated mean differences and corresponding 95% CIs. GA indicates gestational age; P3, Preemie Prep for Parents program.

Pregnant participants in the P3 group scored higher on knowledge of long-term outcomes at 25 weeks than those in the control group (mean percentage correct, 88.5% vs 73.2%; estimated difference, 15.3 percentage points; 95% CI, 8.3-22.5 percentage points; *P* < .001), supporting our primary hypothesis. Considering the PPKQ total score, pregnant participants in the P3 group scored higher than the control group at all 3 time points (Figure 2). Knowledge was consistently higher among P3 group participants for the PPKQ subscales (eTables 3-5 in Supplement 2). Post hoc analyses showed that 57.9% of participants in the P3 group correctly reported the 3 options available for periviable birth treatment in the delivery room (full resuscitation, limited resuscitation, and comfort care) compared with just 3.6% of participants in the control group.

Within the P3 group, participants who used more videos had significantly higher knowledge scores by 34 weeks’ GA. For every 5 more P3 videos watched, PPKQ total scores increased by approximately 2.2 percentage points (95% CI, 1.4-2.9 percentage points).

### Preparation for Decision-Making

**Figure 3.**
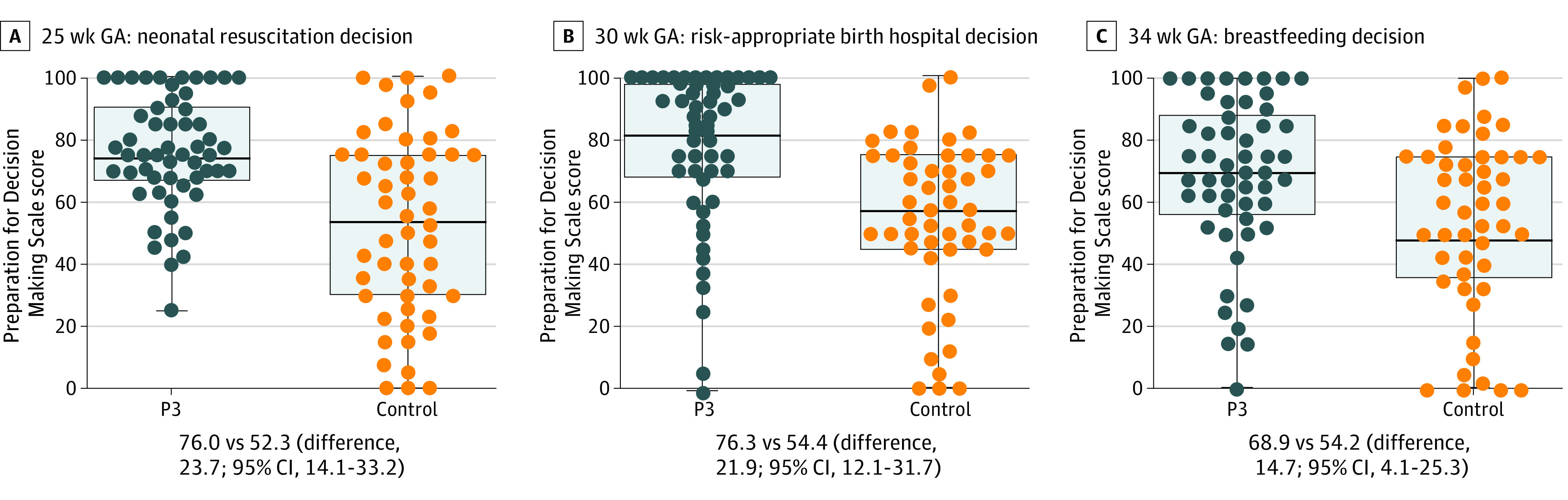
Preparation for Decision Making Scale Scores by Study Group Reported as mean score on a 0- to 100-point scale with the estimated mean differences and corresponding 95% CIs. GA indicates gestational age; P3, Preemie Prep for Parents program.

Compared with the control group, participants in the P3 group reported more preparedness for a neonatal resuscitation decision at 25 weeks (Preparation for Decision Making Scale score, 76.0 vs 52.3; difference, 23.7; 95% CI, 14.1-33.2), for choosing an appropriate birth hospital at 30 weeks (Preparation for Decision Making Scale score, 76.3 vs 54.4; difference, 21.9; 95% CI, 12.1-31.7), and for a decision to breastfeed at 34 weeks (Preparation for Decision Making Scale score, 68.9 vs 54.2; difference, 14.7; 95% CI, 4.1-25.3) (Figure 3; eTable 6 in Supplement 2).

### Anxiety

There were no differences between the P3 and control groups in anxiety at baseline or any subsequent time point (25 weeks: [SE] PROMIS Anxiety scores, 53.8 [1.1] vs 54.0 [1.1]; difference, −0.1; 95% CI, −3.2 to 2.9) (Table 2). Furthermore, neither group experienced a change in anxiety over time.

**Table 2.** PROMIS Anxiety CAT Scores at Each Time Point and Changes in Scores From Baseline, Modeled by Repeated-Measures Linear Model

Assessment time	P3 group	Control group	Estimated difference between groups (95% CI)	Estimated change from baseline (95% CI)	
				P3 group	Control group
Enrollment, estimated mean (SE) score	53.7 (1.0)	54.6 (1.0)	−0.9 (−3.8 to 2.0)	NA	NA
At 25 wk, estimated mean (SE) score	53.8 (1.1)	54.0 (1.1)	−0.1 (−3.2 to 2.9)	0.1 (−1.9 to 2.0)	−0.7 (−2.6 to 1.3)
At 30 wk, estimated mean (SE) score	53.0 (1.0)	55.2 (1.0)	−2.3 (−5.0 to 0.5)	−0.8 (−2.8 to 1.2)	0.6 (−1.4 to 2.6)
At 34 wk, estimated mean (SE) score	53.7 (1.2)	55.4 (1.2)	−1.8 (−5.1 to 1.5)	−0.1 (−2.2 to 2.0)	0.8 (−1.4 to 3.0)

Abbreviations: NA, not applicable; P3, Preemie Prep for Parents; PROMIS Anxiety CAT, Patient-Reported Outcomes Measurement Information System Anxiety Computerized Adaptive Testing.

### Prematurity Preparation and Discussions

At 34 weeks’ GA, participants in the P3 group were more likely than those in the control group to report that the study materials gave them more information than their physician on premature infants (88.5% [46 of 52] vs 66.7% [32 of 48]; estimated difference, 21.8%; 95% CI, 5.9%-37.7%) and on premature labor and delivery (90.4% [47 of 52] vs 58.3% [28 of 48]; estimated difference, 32.1%; 95% CI, 16.0%-48.1%). Although most participants in both groups reported that the study materials led them to ask more questions at their prenatal visits (P3 group, 73.1% [38 of 52]; control group, 70.8% [34 of 48]; estimated difference, 2.3%; 95% CI, −15.4% to 20.0%), pregnant participants in the P3 group were more likely to report that they discussed preterm birth issues with their partner (P3 group, 94.2% [49 of 52]; control group, 64.6% [31 of 48]; estimated difference, 29.6%; 95% CI, 14.7%-44.6%). The groups were similar with respect to creating a plan for preterm labor (P3 group, 84.6% [44 of 52]; control group, 83.3% [40 of 48]; estimated difference, 1.3%; 95% CI, −13.1% to 15.7%).

## Discussion

To our knowledge, this is the first parallel-group randomized clinical trial of preterm birth education in early pregnancy for pregnant persons with risk factors for preterm birth. Compared with a control group receiving ACOG webpage links, participants randomly assigned to the P3 program were more knowledgeable about preterm birth at key time points during pregnancy (25, 30, and 34 weeks’ GA). The P3 participants also reported greater preparedness for making decisions about neonatal resuscitation, birth hospital choice, and breastfeeding. This intervention did not increase participant anxiety.

Prematurity guidance is typically not offered during routine prenatal care for patients with risk factors^10^ because of clinicians’ uncertainty in predicting preterm birth,^30^ concerns of elevating maternal anxiety,^11^ and anticipated disinterest in learning until preterm birth is imminent.^31^ The P3 program seems able to overcome these perceived obstacles. First, our study’s risk factors identified a sample of pregnant persons whose rate of preterm birth (38.7%) was nearly 4 times greater than the general US population rate (10%),^32^ indicating that our identified risk factors estimated preterm birth well and minimized unnecessary exposure. Second, at each follow-up point, anxiety levels were no different from baseline, and the P3 group did not have greater anxiety than the control group. The mean anxiety T scores at enrollment for both groups were higher than the US general population mean, which is in line with other studies reporting higher-than-average levels of anxiety among pregnant persons during the COVID-19 pandemic.^33^ Finally, most pregnant participants used at least half the videos despite preterm birth not being imminent. The P3 program has always been accessible and self-paced; for this randomized clinical trial, we further enhanced the curricula by developing high-quality animations, which have been shown to have broad appeal^15,16^ and educational efficacy among users with lower health literacy.^17^ The short, dynamic videos were likely more engaging than static website links, leading to a high level of engagement among participants.

Participants receiving the P3 program knew more of the information that is recommended by the SMFM, AAP, and ACOG for parents at risk for preterm birth. They also knew more about the potential long-term outcomes associated with preterm birth, which is key information for decision-making that often gets overlooked during traditional counseling.^19^ Although knowledge is not itself behavior change, increased knowledge has corresponded to improvement in self-management for other health concerns, such as diabetes^34^ and heart disease.^35^ Similarly, prenatal education for typical pregnancy has demonstrated improvements in identification of true labor, partner involvement, and maternal anxiety.^36^ Educational aids have been shown to improve patient knowledge and are best suited for preference-sensitive decisions, which include neonatal resuscitation during the periviable period.^37^ Higher levels of patient knowledge have been associated with more realistic perceptions about treatment options, more active patient participation in decision-making, and increased concordance between patient values and treatment choices.^38,39,40,41^

In addition to greater knowledge, the P3 program led to more preparedness for decisions that have implications for maternal and infant health. Pregnant participants in the P3 group felt more prepared to discuss decisions regarding neonatal resuscitation, choosing a risk-appropriate birth hospital, and the decision to breastfeed. Conversations between patients and clinicians in which patients feel informed on perinatal topics typically lead to reductions in decisional conflict and regret.^42,43^ The increased awareness of choice and the treatment options, as offered by the P3 program, may lead to greater readiness to participate in shared decision-making for these choices.^43^

### Limitations

This study has some limitations. The study design—randomized assignment, blinded scoring, and blinded comparison of postintervention recommended knowledge between the P3 and control groups—should produce robust and unbiased results, and our study results demonstrated the P3 program’s ability to improve knowledge and preparedness for key health care decisions. However, we did not assess the program’s effect on actual decisions or health outcomes. Given the comparison of the P3 program to an enhanced standard-of-care control group, we cannot draw conclusions about the specific strengths of the P3 program that caused the improvements in knowledge and preparedness (eg, delivery of GA-specific information, incremental learning, or animated videos). In addition, we were not given permission by ACOG to track participants’ use of the ACOG materials. Although we found no increase in anxiety among the P3 group, the program also did not decrease parental anxiety. However, unlike previous studies^19,44^ demonstrating a reduction in anxiety after education, our intervention targeted a group not yet experiencing a medical threat. Our sample was representative of our clinic’s population but not the larger US general population because most participants held a college degree and were White. Moreover, these subgroups were more likely to use the P3 program. Given the preliminary promise of this intervention, a future trial is needed that is powered to investigate maternal and neonatal health outcomes as well as examine important subgroups most affected by preterm birth.

## Conclusions

Preterm birth is a leading cause of neonatal and infant mortality and childhood morbidity and can have a profound effect on families. Standard prenatal care does not offer anticipatory guidance to pregnant persons at risk of delivering preterm, but the P3 program is a novel intervention that can provide this guidance. In this randomized clinical trial, pregnant persons with access to the P3 program had more knowledge of core competencies and were more prepared to make decisions that affect maternal and infant health, without experiencing increased anxiety. Mobile antenatal prematurity education as offered by the P3 program may provide a unique benefit to parents with preterm birth risk factors.

## Notes

Supplement 1.
Trial Protocol

Supplement 2.
eMethods.

eResults.
**eTable 1.** Distribution of GA at Delivery (n=119) **eFigure.** Distribution of P3 Video Usage **eTable 2.** Video Usage by Participant Characteristics in the P3 Study Arm (n=60) **eTable 3.** Prematurity Knowledge Questionnaire Subscales, % Correct at 25 Weeks **eTable 4.** Prematurity Knowledge Questionnaire Subscales, % Correct at 30 Weeks **eTable 5.** Prematurity Knowledge Questionnaire Subscales, % Correct at 34 Weeks **eTable 6.** Preparation for Decision Making Scale Scores*
eReferences.

Supplement 3.
Data Sharing Statement
