# Supplementary material for: Smartphone-Based Video Antenatal Preterm Birth Education: The Preemie Prep for Parents Randomized Clinical Trial
Source: JAMA Pediatr. 2023 Jul 31;177(9):921–9. doi: 10.1001/jamapediatrics.2023.1586 (PMC10481234; doi:10.1001/jamapediatrics.2023.1586)
Supplement: Supplement 2. — eMethods. eResults. eTable 1. Distribution of GA at Delivery (n=119) eFigure. Distribution of P3 Video Usage eTable 2. Video Usage by Participant Characteristics in the P3 Study Arm (n=60) eTable 3. Prematurity Knowledge Questionnaire Subscales, % Correct at 25 Weeks eTable 4. Prematurity Knowledge Questionnaire Subscales, % Correct at 30 Weeks eTable 5. Prematurity Knowledge Questionnaire Subscales, % Correct at 34 Weeks eTable 6. Preparation for Decision Making Scale Scores* eReferences. [file jamapediatr-e231586-s002.pdf]

Supplementary Online Content

Flynn KE, McDonnell SM, Brazauskas R, et al. Smartphone-based video antenatal preterm birth education: the Preemie Prep for Parents randomized clinical trial. *JAMA Pediatr.* Published online July 31, 2023. doi:10.1001/jamapediatrics.2023.1586

eMethods.
eResults.
eTable 1. Distribution of GA at Delivery (n=119*)
eFigure. Distribution of P3 Video Usage
eTable 2. Video Usage by Participant Characteristics in the P3 Study Arm (n=60)
eTable 3. Prematurity Knowledge Questionnaire Subscales, % Correct at 25 Weeks
eTable 4. Prematurity Knowledge Questionnaire Subscales, % Correct at 30 Weeks
eTable 5. Prematurity Knowledge Questionnaire Subscales, % Correct at 34 Weeks
eTable 6. Preparation for Decision Making Scale Scores*
eReferences.

This supplementary material has been provided by the authors to give readers additional information about their work.

© 2023 Flynn KE et al. *JAMA Pediatrics.*

eMethods.

Development of the P3 Program – Usability Testing

The video scripts were reviewed and refined in collaboration with a parent advisory committee and experts in neonatology, maternal fetal medicine, health literacy, and bioethics. The parent advisory committee included 3 mothers of a preterm infant and 2 fathers of a preterm infant. After the video scripts were finalized, animated videos were created using the software PowToon, allowing for diversity in characters and movements.

Each of the 51 videos underwent remote usability testing with 5-6 lay viewers across the country, including users with a high school diploma or less. Using the survey platform Qualtrics, users watched the videos and were then tested on the learning objective of the video using the corresponding item(s) from the Parent Prematurity Knowledge Questionnaire. We collected feedback regarding the videos' visuals and content for acceptability. Users were given $10 for each video reviewed. User feedback was reviewed by the research team, and if more than one user answered the knowledge question incorrectly or provided negative comments, edits were made and the video retested.

Administration of the P3 Program

Participants in the P3 arm began to receive text messages with links to GA-relevant videos after randomization. The videos were sent at a schedule unique to their GA at enrollment to allow for provision of key content at the most relevant point in the pregnancy. For participants enrolling at 16 or 17 weeks GA, messages did not begin to be sent until the start of 18 weeks GA. Participants received 0-2 text messages per day and participants enrolled at a later GA received more frequent messages to ensure everyone saw the same information. Text messages continued from 18 weeks or enrollment until 34 weeks GA. Some of the videos were sent more than once (up to four times throughout the study), to increase the chance of participants viewing and remembering the most important information. Participants were therefore sent links to the 51 videos through up to 80 text messages, though links ceased if a participant delivered preterm. Participants also had access to a home page housing the full library of videos, giving them the option to "binge watch" videos if they preferred. Study staff monitored video use through software tracking utilization on the website. If less than 10% of the videos sent were watched, staff contacted the participant to ensure there was not a technical error in delivery or viewing.

Health Literacy Assessment

When recruiting in the clinic, information on participants' health literacy was collected using the Newest Vital Sign.[1] In this assessment, the participant is given an ice cream label to review and is tested on their understanding of its words and numbers. However, the measure requires in-person administration. After transitioning to remote recruitment due to COVID-19 restrictions, we began to collect health literacy using the single screening item, "How confident are you in filling out medical forms by yourself?" which has been used to detect inadequate or marginal health literacy.[2]

The Parent Prematurity Knowledge Questionnaire (PPKQ)

With remote data collection, there was the potential that a so-motivated parent may have "cheated" in order to answer PPKQ questions correctly. For all participants, we stressed before each assessment that they should not expect to know all of the answers to the questions, and that the point of the study was for the researchers to understand how to improve the educational tool, thus their honest answers would be most helpful.

Parent Prematurity Knowledge Questionnaire: 25 Weeks
Correct answers are highlighted in yellow.

1. A baby born before __37__ weeks of pregnancy is considered premature.
2. Which method of giving birth is most risky for mothers?
    a. Vaginal birth
    b. Cesarean section
    c. Classic cesarean section
    d. Do not know
3. If you have 10 weeks left to get to your due date, what is your baby's gestational age? __30___ weeks
4. Currently, medical technology is not advanced enough to allow a baby born during or before the __21__ week of pregnancy to survive.
5. A pregnancy that is 23 weeks and 6 days is considered by a doctor to be:
    a. 23 weeks of pregnancy
    b. 24 weeks of pregnancy

© 2023 Flynn KE et al. *JAMA Pediatrics.*

6. What weeks of pregnancy are included in the Gray Zone? __22, 23, 24___
7. Medicines called for tocolytics (toe-ka-lit-iks) can delay delivery for:
    a. 1-2 days
    b. 3-4 days
    c. 5-6 days
    d. Do not know
8. Parents are usually given options for the care of their premature baby born during the Gray Zone of pregnancy. Briefly describe these three options.
    a. Option 1: ____Using medical machines______
    b. Option 2: _________ Comfort care ____________
    c. Option 3: __Limiting use of medical machines__
9. It is best if a premature baby born before 25 weeks of pregnancy is born in a hospital with a:
    a. Level 1 NICU
    b. Level 2 NICU
    c. Level 3 NICU
    d. Do not know
10. Compared to a baby born after 37 weeks, is a baby born before 25 weeks of pregnancy more likely to develop lifelong health problems?
    a. Yes
    b. No
    c. Do not know
11. If the baby is not responding well to treatments, can parents ask doctors to stop using medical machines for their premature baby?
    a. Yes
    b. No
    c. Do not know
12. Do premature girls have a better chance of being healthy than premature boys?
    a. Yes
    b. No
    c. Do not know
13. Can being born premature cause lifelong physical disabilities?
    a. Yes
    b. No
    c. Do not know
14. Compared to a baby born after 37 weeks, is a baby born before 25 weeks of pregnancy more likely to have problems learning?
    a. Yes
    b. No
    c. Do not know
15. Compared to a baby born after 37 weeks, is a baby born before 25 weeks of pregnancy more likely to have brain damage?
    a. Yes
    b. No
    c. Do not know
16. Compared to a baby born after 37 weeks, is a baby born before 25 weeks of pregnancy more likely to have Down's Syndrome?
    a. Yes
    b. No
    c. Do not know
17. Compared to baby born after 37 weeks, is a baby born before 25 weeks of pregnancy more likely to need help with their normal, daily activities for the rest of their life?
    a. Yes
    b. No
    c. Do not know
18. Compared to a baby born after 37 weeks, is a baby born before 25 weeks of pregnancy more likely to be blind?
    a. Yes
    b. No
    c. Do not know
19. Compared to a baby born after 37 weeks, is a baby born before 25 weeks of pregnancy more likely to develop hearing problems?

© 2023 Flynn KE et al. *JAMA Pediatrics.*

    a. Yes
    b. No
    c. Do not know
20. A cervical cerclage is performed if a woman is in preterm labor to help her baby develop more quickly.
    a. True
    b. False
    c. Do not know
21. If parents are not married, the father can legally sign the consent forms for the baby's treatment at birth.
    a. True
    b. False
    c. Do not know
22. If a baby is born during the Gray Zone of pregnancy, doctors would make the decision about what kind of medical care the baby will receive.
    a. True
    b. False
    c. Do not know
23. A premature delivery is very critical, but the mom's support person is still usually allowed to be in the room for a premature birth.
    a. True
    b. False
    c. Do not know
24. Premature babies born as twins or triplets have a better chance of being healthy than premature babies born as a single baby.
    a. True
    b. False
    c. Do not know
25. Two babies born during the 24th week of pregnancy are likely to have the same general level of intellectual abilities.
    a. True
    b. False
    c. Do not know
26. Mother's milk is the best nutrition for a baby, even if the baby is born premature.
    a. True
    b. False
    c. Do not know
27. If you start experiencing signs of preterm labor, you should wait until you know if it is serious before calling a doctor.
    a. True
    b. False
    c. Do not know
28. Most women who go into preterm labor will deliver within the next day or two.
    a. True
    b. False
    c. Do not know
29. Preterm birth is an emergency that cannot be prepared for.
    a. True
    b. False
    c. Do not know
30. All premature babies will have at least mild intellectual limitations.
    a. True
    b. False
    c. Do not know
31. If a premature baby is receiving comfort care, it is not a good idea for parents to change their mind and ask to use medical machines.
    a. True
    b. False
    c. Do not know
32. Premature babies are at greater risk of health problems because they miss out on time to develop in the womb.
    a. True
    b. False
    c. Do not know
33. An intellectual disability would only affect a child's performance in school.

© 2023 Flynn KE et al. *JAMA Pediatrics.*

a. True
b. False
c. Do not know

34. Comfort care is a type of medical care that allows the baby to die naturally.
    a. True
    b. False
    c. Do not know
35. A woman's current week of pregnancy is completely accurate.
    a. True
    b. False
    c. Do not know
36. Steroid shots given to the mother during preterm labor increase the chance of having a healthier premature baby.
    a. True
    b. False
    c. Do not know

Evaluation of Study Materials

De novo questions about study materials covered whether the materials influenced communication with their OB providers or support person, whether they created a plan for preterm labor, and whether their doctor or the study materials provided more information.

P3 group:

1. Because of the text messages, did you ask more questions at your doctor visits?
    a. Yes
    b. No
2. Because of the text messages, did you and your partner discuss premature birth issues?
    a. Yes
    b. No
3. Did you and your family create a plan of what you would do in case you went into preterm labor?
    a. Yes
    b. No
4. Which source provided you more information about premature babies?
    a. My doctor
    b. Texts messages
5. Which source gave you more information about premature labor and delivery?
    a. My doctor
    b. Text messages

ACOG group:

1. Because of the information provided, did you ask more questions at your doctor visits?
    a. Yes
    b. No
2. Because of the information provided, did you and your partner discuss premature birth issues?
    a. Yes
    b. No
3. Did you and your family create a plan of what you would do in case you went into preterm labor?
    a. Yes
    b. No
4. Which source provided you more information about premature babies?
    a. My doctor
    b. Study information
5. Which source gave you more information about premature labor and delivery?
    a. My doctor
    b. Study information

© 2023 Flynn KE et al. *JAMA Pediatrics.*

eResults.

eTable 1. Distribution of GA at Delivery (n=119*)

	Preterm Deliveries				Term Deliveries
GA at Delivery	20-21	22-25	26-32	33-36	37+
Participants, n (%)	2 (1.7)	1 (0.8)	6 (5.0)	37 (31.1)	73 (61.3)
Total, n (%)	46 (38.7)				73 (61.3)

*1 participant was lost to follow-up before delivery

© 2023 Flynn KE et al. *JAMA Pediatrics.*

eFigure. Distribution of P3 Video Usage

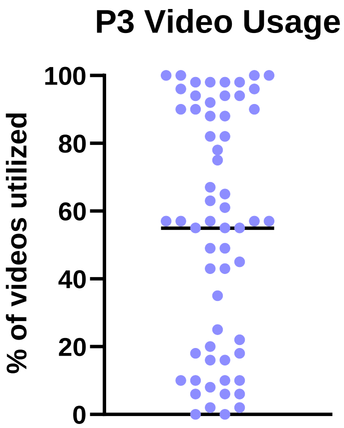

P3 Program Usage

Videos before 25 weeks GA were used on average by 62.5% (SD 36.0%) of pregnant participants, while videos sent between 26 and 30 weeks were watched by 45.4% (39.8%) and videos sent between 31 and 34 weeks were watched by 43.3% (41.8%) of pregnant participants.

© 2023 Flynn KE et al. *JAMA Pediatrics.*

eTable 2. Video Usage by Participant Characteristics in the P3 Study Arm (n=60)

**Characteristic**	**n**	**% Videos Viewed, Mean Estimate (95% CI)**
Race		
White	43	62.7 (52.3, 73.0)
Black	12	25.3 (6.7, 43.9)
Other	5	59.2 (24.8, 93.6)
Ethnicity		
Hispanic or Latino	4	51.0 (1.8, 100)
Not Hispanic or Latino	55	55.4 (45.6, 65.1)
Education		
High school diploma or less	8	25.0 (0, 51.3)
Some college or 2-year degree	20	42.5 (27.0, 57.9)
4-year degree	17	71.4 (56.0, 86.8)
Graduate/professional degree	15	68.8 (51.8, 85.7)
Healthcare Field		
Work in healthcare	20	57.9 (42.3, 73.6)
Do not work in healthcare	39	54.3 (42.5, 66.1)
Marital Status		
Married	38	66.2 (56.3, 76.1)
Not married	21	36.2 (19.9, 52.5)
Living situation		
Living together with father of baby	54	58.0 (48.7, 67.3)
Living separately from father of baby	5	29.0 (0, 78.5)
GA at enrollment		
16 weeks GA	17	59.7 (42.2, 77.3)
17 weeks GA	13	44.9 (22.8, 67.1)
18 weeks GA	2	52.9 (0, 100)
19 weeks GA	10	48.8 (20.5, 77.2)
20 weeks GA	12	67.6 (50.1, 85.2)
21 weeks GA	5	49.0 (0, 100)
22 weeks GA	1	43.1
Pregnancy app already on phone		
Already had pregnancy app	53	56.3 (46.5, 66.2)
Did not already have pregnancy app	7	44.3 (16.0, 72.5)
Daily phone screen time		
0 - <1 hour	3	47.7 (0, 100)
1-2 hours	20	64.0 (50.6, 77.4)
2-3 hours	24	25.1 (35.5, 68.7)
4+ hours	13	47.7 (26.9, 68.4)

© 2023 Flynn KE et al. *JAMA Pediatrics.*

Characteristic	n	% Videos Viewed, Mean Estimate (95% CI)
History of preterm birth		
History of preterm birth	20	41.9 (25.8, 57.9)
No history of preterm birth	40	61.4 (50.5, 72.3)

© 2023 Flynn KE et al. *JAMA Pediatrics.*

Pregnant Participant Prematurity Knowledge

The pattern of greater knowledge in the P3 group was found among all subscales. There were differences between groups for nearly all subscales, with the exceptions of variability in due date estimation at 25 weeks, treatment options at 30 weeks, general prematurity knowledge at 34 weeks, and long-term outcomes at 34 weeks.

eTable 3. Prematurity Knowledge Questionnaire Subscales, % Correct at 25 Weeks

Subscale	Overall	P3	ACOG	Estimated Difference (95% CI)
Long term outcomes (10 items)	81 (20)	89 (14)	73 (23)	15 (8, 23)
Variability in due date estimation (2 items)	80 (30)	83 (30)	77 (35)	6 (-6, 18)
General prematurity knowledge (6 items)	78 (20)	82 (17)	73 (23)	9 (1, 17)
Lowest GA needed for survival (2 items)	21 (30)	33 (35)	9 (20)	24 (13, 35)
Factors influencing preterm birth outcome (4 items)	72 (28)	86 (20)	58 (28)	28 (18, 38)
Treatment options (3 items)	52 (43)	73 (40)	30 (33)	44 (30, 58)
Advocacy (3 items)	69 (33)	86 (23)	53 (30)	33 (22, 44)

© 2023 Flynn KE et al. *JAMA Pediatrics.*

eTable 4. Prematurity Knowledge Questionnaire Subscales, % Correct at 30 Weeks

Subscale	Overall	P3	ACOG	Estimated Difference (95% CI)
Variability in due date estimation (2 items)	88 (25)	92 (20)	84 (25)	8 (0, 17)
General prematurity knowledge (5 items)	70 (14)	72 (12)	66 (14)	6 (1, 11)
Factors influencing preterm birth (7 items)	80 (20)	90 (16)	71 (20)	19 (12, 26)
Treatment options (1 item)	80 (40)	87 (30)	73 (40)	14 (-2, 29)
Short term outcomes (4 items)	87 (20)	90 (15)	83 (23)	8 (1, 16)
Long term outcomes (8 items)	70 (24)	80 (16)	60 (26)	20 (11, 29)
Advocacy (7 items)	75 (20)	86 (13)	66 (20)	20 (13, 26)

© 2023 Flynn KE et al. *JAMA Pediatrics.*

eTable 5. Prematurity Knowledge Questionnaire Subscales, % Correct at 34 Weeks

Subscale	Overall	P3	ACOG	Estimated Difference (95% CI)
General prematurity knowledge (5 items)	81 (18)	83 (16)	78 (20)	6 (-1, 13)
Factors influencing preterm birth (8 items)	80 (21)	89 (14)	70 (23)	20 (12, 27)
Treatment options (1 item)	78 (40)	89 (30)	65 (50)	24 (7, 40)
Short term outcomes (6 items)	71 (20)	78 (15)	63 (22)	15 (7, 22)
Long term outcomes (3 items)	86 (27)	90 (27)	80 (30)	10 (0, 21)
Advocacy (7 items)	80 (20)	88 (16)	71 (20)	17 (10, 24)

© 2023 Flynn KE et al. *JAMA Pediatrics.*

eTable 6. Preparation for Decision Making Scale Scores*

Assessment	Overall	P3	ACOG	Estimated Difference (95% CI)
25 Weeks, mean (SD) *Resuscitation decisions*	64.4 (27.0)	76.0 (18.3)	52.3 (29.5)	23.7 (14.2, 33.1)
30 Weeks, mean (SD) *Birth hospital choice*	65.5 (27.2)	76.3 (24.8)	54.4 (25.1)	21.9 (12.1, 31.7)
34 weeks, mean (SD) *Breastfeeding decision*	61.9 (27.6)	68.9 (25.1)	54.2 (28.5)	14.7 (4.2, 25.3)

*Scores are on a 100-point scale.

© 2023 Flynn KE et al. *JAMA Pediatrics.*

eReferences.

1. Weiss BD, Mays MZ, Martz W, et al. Quick assessment of literacy in primary care: the newest vital sign. *Ann Fam Med*. Nov-Dec 2005;3(6):514-22. doi:10.1370/afm.405
2. Chew LD, Bradley KA, Boyko EJ. Brief questions to identify patients with inadequate health literacy. *Fam Med*. Sep 2004;36(8):588-94.

© 2023 Flynn KE et al. *JAMA Pediatrics.*
