# Supplementary material for: Smartphone-Based Video Antenatal Preterm Birth Education: The Preemie Prep for Parents Randomized Clinical Trial
Source: JAMA Pediatr. 2023 Jul 31;177(9):921–9. doi: 10.1001/jamapediatrics.2023.1586 (PMC10481234; doi:10.1001/jamapediatrics.2023.1586)
Supplement: Supplement 3. — Data Sharing Statement [file jamapediatr-e231586-s003.pdf]

Data Sharing Statement

Flynn. Smartphone-Based Video Antenatal Preterm Birth Education. *JAMA Pediatr*. Published July 31, 2023. doi:10.1001/jamapediatrics.2023.1586

Data
Data available: No
